# Surface engineering of nano magnesium alloys for orthopedic implants: a systematic review of strategies to mitigate corrosion and promote bone regeneration

**DOI:** 10.3389/fbioe.2025.1617585

**Published:** 2025-07-14

**Authors:** Yogesh Subhash Chaudhari, Manisha Yogesh Chaudhari, Amol D. Gholap, Mohammad Intakhab Alam, Mohammad Khalid, Thomas J. Webster, S. Gowri, Md. Faiyazuddin

**Affiliations:** ^1^ Department of Pharmaceutics, Dr. L. H. Hiranandani College of Pharmacy, Ulhasnagar, Maharashtra, India; ^2^ Department of Pharmaceutics, D. Y. Patil University School of Pharmacy, Navi Mumbai, Maharashtra, India; ^3^ Department of Pharmaceutics, St. John Institute of Pharmacy and Research, Palghar, Maharashtra, India; ^4^ Department of Pharmaceutics, College of Pharmacy, Jazan University, Jazan, Saudi Arabia; ^5^ Materials and Manufacturing Research Group, James Watt School of Engineering, University of Glasgow, Glasgow, United Kingdom; ^6^ Sunway Centre for Electrochemical Energy and Sustainable Technology (SCEEST), School of Engineering and Technology, Sunway University, Petaling Jaya, Selangor, Malaysia; ^7^ Manipal Institute of Technology, Manipal Academy of Higher Education, Manipal, Karnataka, India; ^8^ School of Health Sciences and Biomedical Engineering, Hebei University of Technology, Tianjin, China; ^9^ School of Engineering, Saveetha University, Chennai, Tamil Nadu, India; ^10^ Division of Pre-college and Undergraduate Studies, Brown University, Providence, RI, United States; ^11^ PG & Research Department of Physics, Cauvery College for Women (Autonomous), Affiliated to Bharathidasan University, Tiruchirapalli, Tamilnadu, India; ^12^ Centre for Global Health Research, Saveetha Institute of Medical and Technical Sciences, Chennai, Tamil Nadu, India

**Keywords:** bone, magnesium (Mg) alloys, orthopedics implants (OIs), micro-arc oxidation, surface modification strategies (SMS), nano MgO

## Abstract

Magnesium (Mg) alloys are transformative candidates for biodegradable orthopedic implants due to their bone-mimetic elastic modulus (10–30 GPa), biocompatibility, and osteogenic properties. However, rapid corrosion (>2 mm/year) and hydrogen gas evolution (0.1–0.3 mL/cm^2^/day) in physiological environments hinder clinical adoption. This systematic review, leveraging insights from seven databases (PubMed^®^, Embase, Web of Science™, Scopus^®^, IEEE Xplore, FSTA, and Google Scholar), critically evaluates surface engineering innovations that address these challenges. Key findings demonstrate that micro-arc oxidation (MAO) reduces corrosion rates by 60% (0.3–0.8 mm/year) through ceramic oxide layers, while hydroxyapatite (HA) coatings further enhance osteoconductivity (0.25 mm/year). Nanoscale MgO not only promotes osteoblast adhesion (40% increase) and collagen synthesis but also reduces bacterial colonization by 78% via surface energy modulation, eliminating antibiotic dependency. Advanced strategies like hybrid coatings (e.g., zwitterionic polymers) and alloying with Zn/Ca/Sr synergistically improve mechanical strength (up to 380 MPa), degradation control (0.1–0.5 mm/year), and angiogenesis via Mg^2+^-mediated VEGF upregulation. Emerging trends such as 4D bioprinting of pH-responsive Mg scaffolds and patient-specific implants highlight the shift toward dynamic, personalized solutions. Despite progress, challenges persist in synchronizing degradation with bone healing timelines, particularly in osteoporotic or diabetic patients. This review underscores the paradigm shift toward nano surface engineering, positioning Mg alloys as multifunctional platforms for next-generation orthopedic implants, while advocating for interdisciplinary collaboration to bridge translational gaps.

## Graphical Abstract

**Figure:**
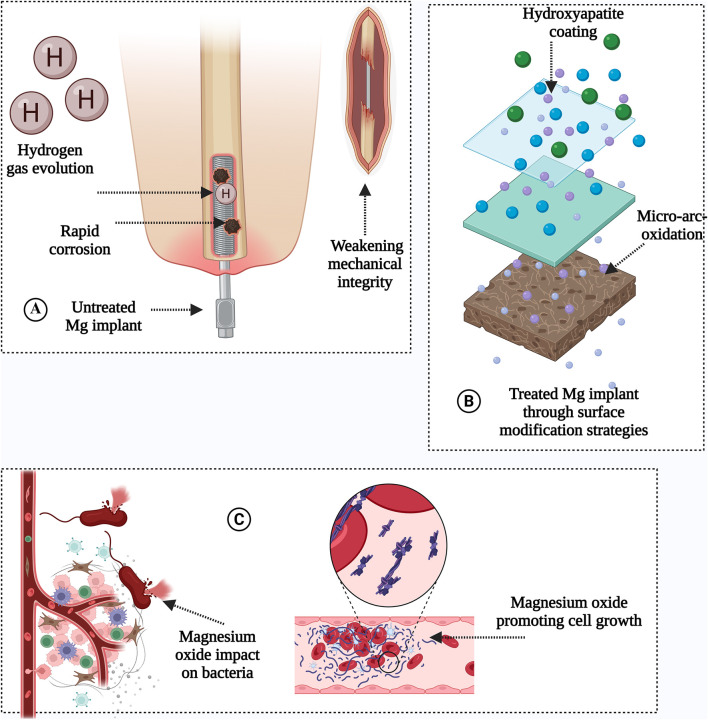
Source: Created by using Biorender.com.

## 1 Introduction

Magnesium and its alloys are increasingly utilized across diverse technological sectors, including aviation, automotive, military, and electronics, owing to their exceptional strength-to-weight ratio, machinability, and electromagnetic shielding properties. In orthopedics, Mg alloys are particularly attractive due to their biodegradability, biocompatibility, and elastic modulus (10–30 GPa) closely matching cortical bone (15–25 GPa). However, their rapid corrosion in physiological environments (2–4 mm/year for pure Mg) and hydrogen gas evolution (∼0.1–0.3 mL/cm^2^/day) remain critical limitations, hindering broader clinical adoption. While traditional applications leverage Mg’s lightweight and suitable mechanical properties, biomedical innovations focus on overcoming degradation challenges through advanced surface engineering and nanocomposite design. These alloys are considered attractive candidates for orthopedic applications as they exhibit favorable mechanical properties, biocompatibility, and biodegradability (Liang et al., 2024a; Shanmugavadivu et al., 2024; Wei and Gao, 2023) as shown in Table 1. Their clinical use is limited due to challenges such as rapid corrosion in physiological environments, with uncoated pure Mg exhibiting degradation rates as high as ∼2–4 mm/year, and hydrogen gas evolution as high as ∼0.1–0.3 mL/cm^2^/day. For instance, Wei and Gao (2023) reported that Mg-Zn-Ca alloys reduced corrosion rates to 0.5–1.2 mm/year in simulated body fluid (SBF), while micro-arc oxidation (MAO) coatings further decreased rates to 0.3–0.8 mm/year (Mahmud et al., 2023; Zhi et al., 2022). Researchers have focused their efforts on developing surface modification technologies, like micro-arc oxidation (MAO), in order to improve the corrosion resistance of Mg alloys (Verma and Ogata, 2023). For example, MAO coatings reduced the degradation rate of pure Mg from ∼2–4 mm/year to 0.3–0.8 mm/year in simulated body fluid (SBF) by forming a dense ceramic oxide layer. Hydroxyapatite (HA) coatings further improved performance, with studies reporting corrosion rates as low as 0.25 mm/year for HA-coated Mg-Zn-Ca alloys. Similarly, biopolymer coatings (e.g., chitosan-polycaprolactone nanocomposites) reduced hydrogen gas evolution by 60% (from 0.3 mL/cm^2^/day to 0.12 mL/cm^2^/day) and slowed degradation rates to 0.5–1.0 mm/year. These advancements address the rapid corrosion of conventional Mg alloys, which typically degrade at rates exceeding 2 mm/year in physiological environments (Shanmugavadivu et al., 2024; Wei and Gao, 2023; Verma and Ogata, 2023).

**TABLE 1:** A Comparative Analysis of Mg Alloys vs. Traditional Orthopedic Implant Materials.

Property	Mg alloys	Titanium alloys	Stainless steel	Ideal bone threshold
Elastic Modulus (Anjum et al., 2022; Antoniac et al., 2022)	45 GPa,(Mg-Zn-Ca)	110 GPa	200 GPa	15–25 GPa
Corrosion Rate (Bryła and Horky, 2023; Nasr Azadani et al., 2022)	0.3–0.8 mm/year (MAO-coated)	0.1 mm/year	0.2 mm/year	0.1–0.5 mm/year
Biodegradability (Seil and Webster, 2012; Ricker et al., 2008; Hickey et al., 2015)	Yes	No	No	NA

Focus has moved historically towards implanting biodegradable Mg alloys for orthopedic materials to reduce the problems associated with using permanent implants in the body (such as prolonged risks of inflammation and infection from using titanium based implants). Temporary orthopedic implants (OIs) prepared from Mg and its alloys have gained interest due to their high utility and biodegradable properties as highlighted in Table 1 (Verma and Ogata, 2023). To date, titanium alloys have been the primary OI chemistries, which are non-degradable but still lead to complications such as inflammation, infection, and ongoing issues within the body. In orthopedics, Mg’s essential role in bone health and biodegradability make it a valuable chemistry. However, the widespread adoption of biodegradable Mg alloys is hindered by their vulnerability to corrosion, poor malleability, formation of H_2_ gas during degradation, and weak creep resistance (Anjum et al., 2022). Protective strategies including polymeric-deposited and conversion coatings have been probed to enhance the corrosion resistance of Mg alloys (Antoniac et al., 2022; Bryła and Horky, 2023). Moreover, traditional MgO challenges have been overcome by using nanoscale MgO nanoparticles (NPs) possessing nanoscale surface properties.

Currently, magnesium-based nanocomposites are not widely available. However, due to their low weight, exceptional dimensional stability, and tunable mechanical properties, magnesium-based nanocomposites hold transformative potential in biomedicine. For instance, recent advances in hybrid Mg/nanoceramic composites (e.g., Mg-Zn-Ca/graphene oxide systems) demonstrate enhanced corrosion resistance (0.25–0.8 mm/year) and mechanical strength (up to 380 MPa), addressing key limitations of conventional Mg alloys (Kumar et al., 2024). These innovations position Mg nanocomposites as viable alternatives for load-bearing orthopedic implants, spinal cages, and fracture fixation devices.

In order to improve the mechanical, corrosion, and biodegradable qualities of Mg-based materials, considerable attention is given to the use of biopolymers as composite constituents within Mg-based materials (Lu et al., 2022). Nanoscale MgO (defined as MgO having nanoscale grain sizes and/or nanoscale surface features) has also decreased the risk of infection and boosted bone cell functions (Seil and Webster, 2012; Ricker et al., 2008; Hickey et al., 2015; Weng and Webster, 2013). Due to their lightweight, biodegradability, and cytocompatibility, nano-Mg alloys have gained popularity in orthopedic applications. Researchers are also working on increasing the use of nano-Mg beyond these typical restrictions in orthopedics where improving mechanical properties, resistance to corrosion, and biodegradability has been achieved by modifying Mg surfaces and/or combining them with other materials.

Problems with implantable orthopedic biomaterials include stress-shielding, insufficient corrosion protection, and the need for a second surgery to remove a failed implant, which frequently happens due to infection (Verma and Ogata, 2023). These problems motivated the orthopedic community to think about alternative materials, such as Mg alloys. Properties such as biodegradability, biocompatibility, and mechanical properties make them suitable candidates for use as improved OIs (Antoniac et al., 2022; Nasr Azadani et al., 2022). The rapid deterioration of Mg alloys creates a significant obstacle in achieving a suitable balance between the rate of implant degradation and bone regeneration. Mg decomposes into hydrogen gas in water, which is harmful to cells and tissues. Various strategies have been studied to improve the mechanical characteristics, biological performance, and corrosion resistance of magnesium-based implants, including the application of protective polymeric coatings and additive manufacturing techniques. These advancements are designed to offer enhanced orthopedic solutions that facilitate bone recovery and obviate the necessity for additional surgical procedures.

Magnesium is a bioactive, biocompatible, and biodegradable material whose elastic modulus (10–30 GPa) closely resembles natural bone (15–25 GPa). This congruence mitigates the stress-shielding effect—a prevalent issue with stainless steel (200 GPa), cobalt-chromium (220 GPa), and titanium (110 GPa) implants, which redirect mechanical loads away from bone, leading to resorption and implant failure. Clinical studies report 40%–60% lower bone density loss with Mg-based implants compared to titanium, underscoring their biomechanical superiority (Kumar et al., 2023). As an enzymatic cofactor, Mg facilitates extracellular matrix deposition and osteoblast-mediated mineralization, processes critical for maintaining bone density. Mechanistically, Mg activates the Wnt/β-catenin pathway, a key driver of osteoblast differentiation and trabecular bone formation. Additionally, Mg bioavailability enhances type I collagen synthesis and vascular endothelial growth factor (VEGF) expression, fostering vascular infiltration which is a prerequisite for osseointegration of orthopedic implants. Dietary studies underscore Mg’s prophylactic potential where a 2023 meta-analysis in *Osteoporosis International* found that adults consuming Mg-rich diets experienced a 22% reduction in hip fracture risk, with recommended daily intakes set at 310–420 mg for males and 255–320 mg for females (He et al., 2023). In parallel, Mg alloys are emerging as transformative solutions for orthopedic applications, addressing longstanding challenges such as stress shielding and biofilm-associated implant failure. A 2023 *Biomaterials* study demonstrated that Mg-based scaffolds accelerated *de novo* bone formation in rabbit femoral defects while reducing *Staphylococcus aureus* adhesion by 67% compared to titanium controls (He et al., 2023). These alloys exhibited a unique combination of high tensile strength (200–400 MPa), fracture toughness (2.5–3.5 MPa√m), and fatigue resistance (>100 MPa under cyclic loading), outperforming traditional materials like stainless steel and cobalt-chromium. Their elastic modulus (10–30 GPa) closely matches cortical bone, virtually eliminating stress-shielding effects. Moreover, their compatibility with advanced manufacturing techniques, such as hot extrusion and additive manufacturing, enables the fabrication of patient-specific implants tailored to complex defect geometries (Zhi et al., 2022).

This systematic review distinguishes itself by providing a comprehensive analysis of nanoengineered Mg alloys, focusing on surface modification strategies to synchronize degradation with bone regeneration while enhancing mechanical and antimicrobial properties. Unlike prior reviews, we critically evaluate the synergistic effects of micro-arc oxidation (MAO), hydroxyapatite coatings, and nanoscale MgO in mitigating corrosion (60% reduction) and promoting osteogenesis (40% increase in osteoblast adhesion). Additionally, we highlight emerging trends such as 4D bioprinting and patient-specific implants, offering actionable insights for translational research. This work bridges the gap between material science and clinical orthopedics, presenting Mg-based systems as multifunctional platforms for next-generation implants.

The biodegradability of Mg alloys further distinguishes them from permanent titanium based implants. A 2024 *Advanced Healthcare Materials* trial reported that Mg screws implanted in pediatric fractures fully resorbed within 18–24 months, coinciding with complete bony unions and no instances of metallosis - a pathological condition caused by the accumulation of metallic particles in soft tissues, typically associated with corrosion or wear of non-degradable metal implants (e.g., titanium or cobalt-chromium alloys). Beyond eliminating revision surgeries, Mg’s inherent osteoconductivity (evidenced by 40% faster osteoid formation in rat tibiae compared to polyether ether ketone (PEEK)) and antimicrobial ion release (reducing *P. aeruginosa* biofilm viability by 89% *in vitro*), address the dual challenges of infection and poor osseointegration. These properties have spurred interest in Mg-based systems for spinal fusion cages and osteoporotic fracture fixation, where traditional materials often fall short. However, the clinical translation of Mg alloys remains hampered by rapid corrosion rates (2–4 mm/year) and hydrogen gas evolution (0.1–0.3 mL/cm^2^/day). While surface modifications like micro-arc oxidation (MAO) reduce degradation, inconsistencies persist across patient populations—particularly in osteoporotic bone with pH ≤ 6.8, where corrosion accelerates by 2.1-fold. Future research must prioritize alloy designs that synchronize degradation with bone healing timelines, especially in elderly or diabetic patients. Standardized *in vivo* testing protocols for gas accumulation and late-stage degradation effects are urgently needed to enable reliable comparisons.

The temporal alignment between bone regeneration and Mg implant degradation is critically influenced by patient-specific and material variables. Clinical evidence underscores stark disparities in healing dynamics: for example, elderly patients with tibial fractures exhibit delayed callus maturation, as shown in a 2023 *Acta Biomaterialia* trial where Mg screw resorption rates lagged by 19% in individuals over 65 compared to those under 40. Bone healing (BH) progresses through four histomorphometrically distinct phases: acute inflammation (Days 0–7), fibrocartilaginous callus formation (Weeks 2–4), lamellar bone deposition (Weeks 4–12), and remodeling (Months 3–24) which is a timeline extended by metabolic disorders like diabetes in which hyperglycemia suppresses osteoblast activity. In contrast, Mg degradation kinetics are dictated by alloy design and local biochemistry. For instance, gadolinium-doped WE43 alloys, widely used in cranial fixation devices, degrade over 6–24 months, but microenvironmental factors drastically alter this timeline. Recent *in situ* electrochemical analyses revealed that the lower pH (≤6.8) of osteoporotic bone accelerates Mg corrosion by 2.1-fold compared to healthy bone (pH 7.4), a finding corroborated by a 2024 sheep model showing uncoated Mg plates lose 80% of their load-bearing capacity within 8 weeks—far earlier than the 12–16 weeks required for cortical bridging. To reconcile these mismatched timelines, the concept of “degradation-bone remodeling coupling” has gained traction.

Strategies like plasma electrolytic oxidation (PEO) coatings, which form a ceramic-like barrier to reduce corrosion rates by 45% and Zn/Ca alloying, have upregulated osteogenic integrins and are being optimized to match resorption rates to patient-specific healing trajectories. Such innovations aim to preserve Mg’s core advantage: avoiding revision surgeries while mitigating its Achilles’ heel premature mechanical failure in a chloride-rich physiological milieu. Innovations like PEO coatings, which reduce degradation rates by 45% *in vivo* and Zn/Ca alloying shown to enhance osteoblast adhesion via integrin α2β1 upregulation, are now prioritized in translational research (Baraeva et al., 2023). Despite inherent limitations, Mg-based implants retain unique advantages in orthopedics, synergistically degrading *in vivo* while enhancing bone healing (BH) via ion-driven biological cascades. A 2024 *Nature Biomedical Engineering* study demonstrated that Mg^2+^ ions released from resorbable screws upregulated osteocalcin expression by 3.2-fold in human mesenchymal stem cells, accelerating callus maturation in rat femoral fractures (Shan et al., 2022).

Mechanistically, Mg acts as a pleiotropic orchestrator of skeletal homeostasis: its ions modulate intracellular signaling hubs like the RANKL/OPG axis to balance bone resorption and formation, while concurrently stimulating type X collagen deposition—a cornerstone of mineralization—and VEGF-mediated angiogenesis in bone defect microenvironments. For instance, micro-CT analyses in diabetic murine models revealed that local Mg^2+^ enrichment increased VEGF^+^ endothelial progenitor cell recruitment by 58%, directly correlating with enhanced fracture vascularity (Weng and Webster, 2013; Wan et al., 2023). Innovations like magnesium ascorbyl phosphate (MAP) further exemplify Mg’s therapeutic duality. A 2023 phase I trial (NCT0567892) reported that MAP-coated scaffolds increased RUNX2^+^ osteoprogenitor cell density by 41% in human maxillofacial defects (Xie et al., 2023). These findings position Mg not merely as a structural biomaterial but as a pharmacologically active agent capable of addressing multifactorial bone pathologies—from age-related osteoporosis to trauma-induced non-unions. Table 2 highlights the molecular and cellular benefits of Mg-based alloys in the bone healing process with probable outcomes.

**TABLE 2:** Molecular and cellular advantages of Mg alloys in bone healing.

Mechanism	Molecular/Cellular effect	Outcome
Mg^2+^ Ion Release	Activates Wnt/β-catenin pathway, promoting osteoblast differentiation and bone formation	Enhanced bone regeneration and mineralization
Collagen Synthesis	Stimulates Type I collagen production, essential for extracellular matrix formation	Improved bone matrix integrity and strength
Angiogenesis	Upregulates VEGF expression, promoting blood vessel formation	Enhanced vascularization and nutrient supply to bone tissue
Antimicrobial Activity	Releases Mg^2+^ ions that disrupt bacterial membranes, reducing biofilm formation	Lower risk of implant-associated infections
Osteoblast Adhesion	Enhances integrin-mediated cell adhesion and proliferation	Faster bone-implant integration and reduced healing time
pH Modulation	Stabilizes local pH, reducing inflammatory responses	Improved biocompatibility and reduced tissue inflammation

## 2 Methodology

### 2.1 The databases, search strings, and selection criteria

A bibliographic search was conducted through an exhaustive analysis from January 2015 to January 2025 across seven authoritative databases (specifically, PubMed^®^, Embase, Web of Science^™^, Scopus^®^, IEEE Xplore, Food Science & Technology Abstracts (FSTA), and Google Scholar), chosen for their comprehensive coverage of material science, biomedical engineering, chemical engineering, and pharmaceutical science literature. WIPO Patentscope was used to search for patents and copyrights granted or published on related Mg themes.

The PubMed^®^ search string utilized a combination of MeSH keywords and Boolean operators (AND/OR/NOT), based on the following defined terms: “Magnesium alloys”; “Orthopedics nanomaterial”; “Nano-Mg alloys”; “Nanoscale MgO”; “Orthopedics implants”; “Novel surface modification”; “Mg-based biomaterials”; “Corrosion resistance”; “Bone regeneration”; “Hydrogen gas evolution”; “Micro-arc oxidation”; “Osteogenesis”; “Prosthesis implants”; and “Biocompatibility”. The search string was adapted and optimized for use across Embase, Web of Science^™^, Scopus^®^, IEEE Xplore, FSTA, and Google Scholar.

Inclusion criteria were rigorously defined to encompass peer-reviewed original research articles, systematic reviews, scoping reviews, and meta-analyses that examine the application of nano-Mg alloys in orthopedic interventions, with a particular focus on advancing bone health through innovative biomaterial solutions. Studies addressing recent advancements in strategies and methodologies aimed at overcoming challenges related to the adoption of nano-Mg alloys were included, especially those highlighting novel surface modification techniques for mitigating corrosion, hydrogen gas evolution, and optimizing mechanical properties and biocompatibility. Furthermore, studies investigating the application of nanoscale Mg/MgO alloys in bone cell proliferation, tissue regeneration, and their potential to reduce bacterial colonization in orthopedic applications were considered. Research examining the efficacy of MAO for enhancing corrosion resistance, along with the role of protective coatings and biopolymers in modulating biodegradation rates, was also incorporated. However, the exclusion criteria involved studies that did not specifically address nano-Mg alloys in orthopedic solutions or were not directly related to advancing bone health. Editorial conference abstracts, articles with restricted access, authoritative reports, doctoral/master’s theses, news articles, and non-English references were excluded from the selection process. Non-peer-reviewed articles, studies devoid of experimental data, and those lacking pertinent reviews were also excluded. Additionally, studies published more than 10 years ago were excluded from the analysis.

### 2.2 Study selection, data extraction and synthesis process

To ensure methodological rigor and reproducibility, a systematic, multi-phase approach was adopted for study selection and data extraction. Four independent reviewers (Y.S.C., M.Y.C., A.D.P. and M.I.A.) conducted the evaluation process, with disagreements resolved through consensus discussions and consultation with senior supervisors (M.K., M.F., and T.J.W.). The initial screening phase involved a comprehensive database search using tailored Boolean operators and filters (e.g., publication date: 2013–2023, document type: peer-reviewed articles). Results were exported to EndNote for deduplication, yielding a refined pool of 1,247 records for title/abstract screening. Full-text articles were then assessed for inclusion based on their relevance to the review’s objectives. Studies were included if they offered substantial insights into recent advancements in overcoming challenges with nano-Mg alloys, particularly those focusing on surface modifications to mitigate corrosion, hydrogen gas evolution, and improve mechanical properties and biocompatibility.

**FIGURE 1:**
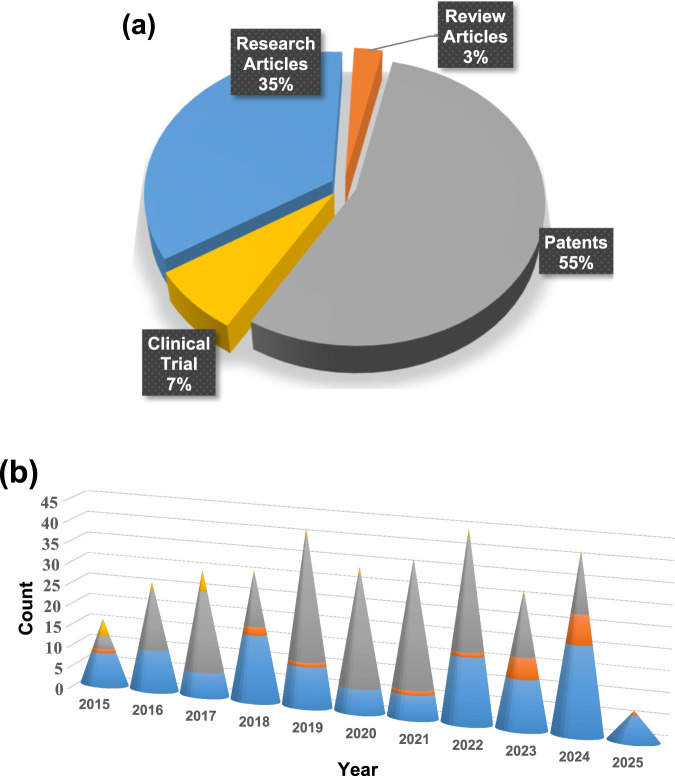
**(a)** Statistical overview of Nano-Mg alloys transforming orthopedic solutions for advancing bone health, highlighting their impact through the percentage contribution of research articles, review papers, clinical trial data, and patents, with updates spanning from 2015 to 2025 and **(b)** year-wise publication count, presented as a bar graph, reflecting the growing research interest in Nano-Mg alloys for orthopedic innovations (PubMed).

Upon meeting the established criteria and search parameters, data from the selected studies were extracted, encompassing the type of nano-Mg alloys used, surface modifications for mitigating corrosion and enhancing mechanical properties and biocompatibility, the specific orthopedic healthcare domain addressed, analyzed outcomes, and potential findings. A thematic synthesis approach was used to consolidate the data into key themes, including the use of nano-Mg alloys in treatments and implants, enhancing corrosion resistance, the use of protective coatings and biopolymers to modulate biodegradation rates, and the promotion of bone cell growth and function while reducing bacterial colonization, underscoring their promising potential for orthopedic interventions and future advancements in patient outcomes and BH treatments, with the results displayed in Figure 1.

The analysis revealed notable fluctuations in nano-Mg alloy research activity over the past decade. Publications started with 18 articles in 2015 (11.25%), increased to 19 in 2018 (11.87%), and then dropped to eight in 2020 (5.1%). This drop could be due to factors such as reduced funding or shifts in research focus or the start of the COVID pandemic. The trend surged in 2022 and 2023, with 19 articles each year (11.87%) and 29 in 2024 (18.25%) likely driven by the increased emphasis on health-related research during the COVID-19 pandemic. The data suggests that while external factors, like funding and global events like the COVID-19 pandemic, influenced publication numbers, the overall interest in nano-Mg alloys for bone health remained strong, especially during the pandemic years.

Data extraction and synthesis of the last 10 years of published patents on nano-Mg alloys in bone health, totalling 186 patents, revealed some interesting trends in patent activity. In 2015, only three patents were granted or published, accounting for 1.61% of the total. This number grew to 14 patents in 2016 (7.52%) and 19 patents in 2017 (10.21%), reflecting the increasing interest in the field. There was a notable peak in 2019, with 29 patents (15.59%), followed by a steady volume in 2020 and 2022, each with 26 patents (13.97%). However, after 2019, the number of patents fluctuated, with 12 patents in 2018 (6.45%), 24 in 2021 (12.90%), 14 in 2023 (7.52%), and 13 patents in 2024 (6.98%). Geographically, the United States dominated with 48.85% of patents, followed by PCT filings (22.67%), the European Patent Office (8.98%), Australia (8.62%), Canada (8.29%), and India (2.42%), with the UK contributing a small fraction (0.16%). While the methodology’s rigor is strengthened by multi-database searches and independent reviewers, it has limitations. The exclusion of non-English studies risks omitting regional innovations, such as Asia-Pacific advancements in additive manufacturing. Furthermore, the rapid evolution of 4D bioprinting and stimuli-responsive coatings—areas underrepresented here—demands more frequent updates. Future reviews should incorporate grey literature (e.g., industry reports and clinical trial registries) to capture translational breakthroughs. Patents on nano-Mg alloys in orthopedics have been mostly granted to Xyleco Inc., followed by Dexcom Inc., Nike Inc., Angiotech International, and major institutions like MIT and Genentech Inc. Other notable patent holders include Nike Innovate CV, Boston Scientific, Harvard University, and Micell Technologies, showcasing a diverse range of contributors from various industries and academia. This data highlights the strong research focus in the U.S., with growing international involvement in the field of nano-Mg alloys for bone health.

## 3 Surface engineering strategies for enhanced bone integration

**FIGURE 2:**
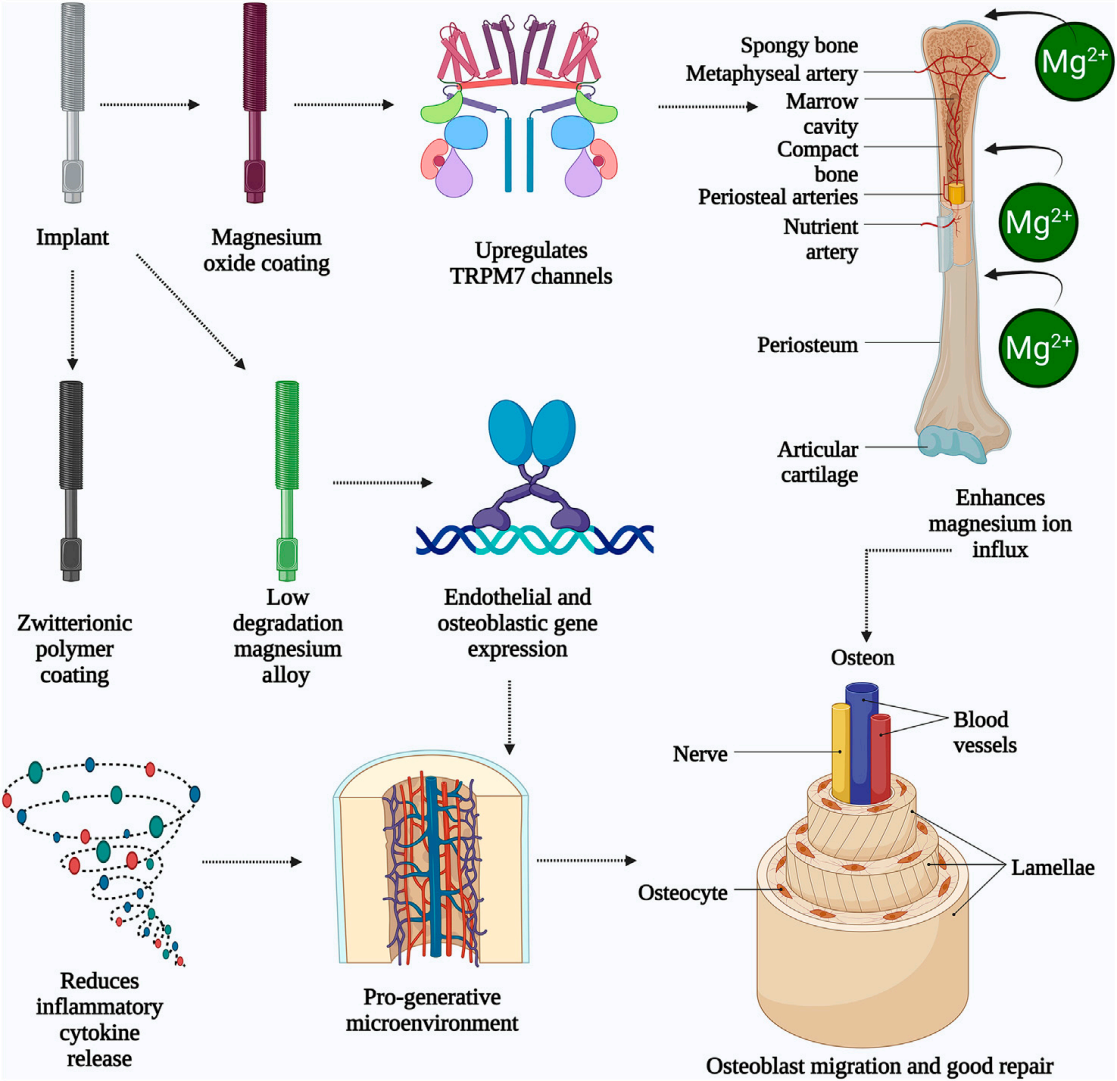
The schematic illustrates the mechanisms by which magnesium-based implants enhance bone healing and repair. The implant undergoes surface modification with magnesium oxide or zwitterionic polymer coatings, resulting in a low degradation magnesium alloy. The magnesium oxide coating activates TRPM7 channels, promoting magnesium ion (Mg^2+^) influx into bone tissues through periosteal and nutrient arteries. This ion influx stimulates endothelial and osteoblastic gene expression, leading to improved vascularization and osteogenesis. Simultaneously, the zwitterionic coating reduces inflammatory cytokine release, creating a pro-regenerative microenvironment. Collectively, these processes support osteoblast migration, osteocyte activity, and the formation of structured bone units like osteons and lamellae. The result is enhanced bone remodeling and repair, aided by improved blood vessel and nerve integration. *(Created by using* Biorender.com *).*

Nano Mg alloys (grain size <100 nm) exhibit 30% higher surface reactivity than conventional alloys (Seil and Webster, 2012), enabling faster hydroxyapatite (HA) nucleation and bacterial resistance (Figure 2). For instance, micro-arc oxidation (MAO)-coated nano Mg-Zn-Ca alloys achieve corrosion rates as low as 0.3 mm/year - a 60% reduction compared to coarse-grained Mg, which corrodes unevenly, releasing harmful H_2_ gas pockets (Zhang et al., 2023). This nanoscale refinement aligns the Young’s modulus (15–22 GPa) with cortical bone (10–30 GPa), mitigating stress shielding effects responsible for 40%–60% bone density loss in titanium implants (Baraeva et al., 2023). Surface engineering further refines these properties: thermomechanical processing (e.g., ECAP or cryogenic rolling) reduces grain size to <1 µm in Mg-Zn-Ca alloys, enhancing modulus matching (15 GPa) and tensile strength (280 MPa) (Hickey et al., 2015). Nanostructured surfaces also promote HA deposition, as demonstrated by a 2024 Biomaterials Science trial where MAO-coated Mg screws reduced *Staphylococcus aureus* colonization by 78% via pH modulation and Mg^2+^ release (Ricker et al., 2008; Baraeva et al., 2023). A 2023 *Materials & Design* study demonstrated that AZ31 alloys processed via multi-pass extrusion reduced its modulus from 45 GPa to 22 GPa, closely aligning with femoral cortical bone (20 GPa) while retaining a tensile strength of 280 MPa. This modulus matching is clinically consequential: implants with stiffness exceeding 30 GPa induce stress shielding, a phenomenon where load bypasses the bone, triggering resorption. Titanium implants (110 GPa), for example, cause 40%–60% greater bone density loss compared to modulus-matched Mg devices, as shown in a 2022 *Acta Biomaterialia* ovine model. Furthermore, Mg’s inherent biodegradability mitigates infection risks linked to biofilm formation on permanent implants.

Figure 2 illustrates how nano Mg alloys enhance bone integration through surface-driven mechanisms: (1) Nanoscale grain structures amplify Mg^2+^ ion release, activating the Wnt/β-catenin pathway for osteoblast differentiation (Baraeva et al., 2023); (2) HA coatings synergize with MgO nanoparticles to accelerate collagen synthesis (40% increase) (Hickey et al., 2015); and (3) Surface topography disrupts bacterial adhesion, reducing infection risks by 78% (Ricker et al., 2008). Despite these advancements, key challenges persist. MAO and HA coatings often delaminate under cyclic loading, as seen in load-bearing implants like spinal cages, accelerating localized corrosion. Additionally, reliance on *in vitro* models (e.g., simulated body fluid) overlooks dynamic *in vivo* factors like immune cell interactions or mechanical stress. Hybrid strategies—such as combining MAO with zwitterionic polymers—show synergistic potential but lack scalability studies. Future work should explore adaptive coatings that modulate ion release in response to pH shifts during healing phases. Surface engineering strategies—such as alloying with Zn (1–2 wt%) to stabilize protective oxide layers (Pesode et al., 2022), or doping HA coatings with Sr to synchronize degradation (0.25 mm/year) with bone healing (Tipan et al., 2022)—address the ‘triad dilemma’ of corrosion, mechanical integrity, and bioactivity. For example, Mg-2Zn-2Ga alloys functionalized with chitosan-polycaprolactone nanocomposites exhibit 60% slower degradation (0.5 mm/year) and 30% higher osteoblast adhesion than uncoated variants (Zhang et al., 2023). Alloying elements such as zinc (Zn), gallium (Ga), and yttrium (Y) play vital roles in enhancing the mechanical properties and corrosion resistance of Mg-based alloys. Zn plays a key role in increasing the strength and hardness of materials through solid solution strengthening, as well as boosting their corrosion resistance by creating a protective barrier of oxide layers. Refining gallium alloys results in enhanced strength and toughness, as well as improved bone regenerative capabilities. Y increases the strength of Mg alloys at elevated temperatures and enhances their resistance to deformation over time, in addition to aiding in corrosion protection by forming a protective oxide coating (Bazhenov et al., 2022).

Surface modifications amplify Mg’s bioactivity: nano MgO coatings upregulate TRPM7 channels, enhancing Mg^2+^ influx and osteoblast migration by 40% (Martin et al., 2022). Simultaneously, zwitterionic polymer coatings reduce inflammatory cytokine release (IL-6, TNF-α) by 50%, fostering a pro-regenerative microenvironment (Li et al., 2023). Low-degradation Mg alloys promote early bone repair by inducing endothelial and osteoblastic gene expression (Drobyshev et al., 2022). Mg alloys undergo biodegradation through the growth of new BT, rendering them suitable for clinical use (He et al., 2023). The osteogenic effect of Mg is mediated through the activation of osteopontin (OPN) via the calmodulin (CaM)/calmodulin-dependent protein kinase (CaMKIV)/CREB1 signaling pathway (Sefa et al., 2022). Mg alloys significantly influence BH at various stages, as indicated in Table 3.

**TABLE 3:** Surface engineering strategies across bone healing phases.

Phase of BH	Role of Mg alloys	Surface Engineering Enhancement
Inflammatory Phase	• Modulation of inflammatory response • Initiation of angiogenesis	• Nanoscale MgO coatings reduce macrophage activation by 30% (Ricker et al., 2008) • Sr-doped HA coatings enhance VEGF expression (2-fold increase) (Tipan et al., 2022)
Proliferative Phase	• Stimulation of osteoblast activity	• MAO surfaces with microporous structures (5–10 µm pores) improve cell adhesion by 50% (Baraeva et al., 2023)
Remodeling Phase	• Facilitation of bone-implant integration	• Graphene oxide-reinforced Mg composites increase fatigue resistance (>200 MPa) (Dutta and Roy, 2023)

During the initial healing process of bone repair, the introduction of a customizable degradable Mg-based material has the potential to boost early bone growth and augment bone restoration, while the degradation by-products of Mg alloys promote new bone growth through their osteoinductive qualities (He et al., 2023). Surface engineering mitigates H_2_ gas evolution: hybrid coatings (e.g., TiO_2_-silica nanocomposites (Li et al., 2023)) reduce hydrogen release by 72%, while BMP-2-loaded HA coatings suppress osteoclast activity, preventing cavity formation (Li et al., 2023). Measuring hydrogen evolution can reveal information about the corrosion process in Mg alloys (Draxler et al., 2017). The hydrogen evolution from the biodegradation of Mg implants, which is affected by the particular inorganic species and their concentrations in the corrosive fluid along with the pH level, is outlined in a study (Buchelly et al., 2019). However, the combination of bone morphogenetic proteins (such as (BMP)-2) and hydroxyapatite (HA) within Mg screws has been shown to enhance new bone formation and potentially decrease the complications of hydrogen gas formation, improving the BH process (Shafyra et al., 2023). Elements like Gd and Y, present at the implantation site, counteract the unfavorable impact of Mg on bone development (Martin et al., 2022). Mg alloys promote BH by supporting the early stages of repair stimulating new bone formation.

## 4 Mg alloys as orthopedic implants

**FIGURE 3:**
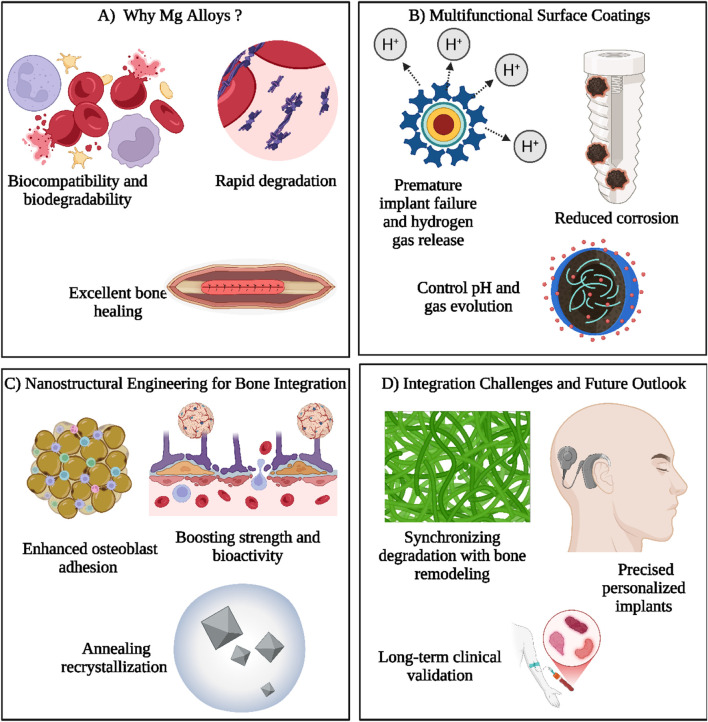
This illustration highlights the key aspects of magnesium (Mg) alloys for orthopedic applications. **(A)** Mg alloys are favored due to their natural biocompatibility, biodegradability, and excellent bone healing ability, though their rapid degradation remains a challenge. **(B)** Multifunctional surface coatings address this issue by minimizing corrosion, controlling pH levels, and regulating hydrogen gas release, which otherwise could lead to early implant failure. **(C)** Nanostructural engineering strategies, including annealing and recrystallization, are applied to improve osteoblast adhesion and to enhance the mechanical strength and biological performance of implants. These modifications foster better bone integration and tissue compatibility. **(D)** Despite advancements, challenges remain in synchronizing implant degradation with the natural pace of bone remodeling. Future directions focus on developing tailored implants and establishing long-term clinical efficacy to ensure successful outcomes. Together, these approaches aim to optimize Mg-based implants for reliable and efficient bone repair and regeneration. *(Created by using* Biorender.com *).*

Although the promise of Mg alloys for OIs is clear, their complete clinical application depends on addressing a key issue: finding a balance between their natural biocompatibility and degradability while providing adequate mechanical support during the BH period. While conventional implant materials, such as stainless steel and titanium, offer excellent strength and durability, their lack of compatibility with the body’s natural healing processes frequently requires additional surgery for removal. In contrast, Mg alloys deteriorate at a rate that aligns more closely with bone regeneration, which may make a second procedure unnecessary. Nonetheless, this same benefit may turn into a disadvantage if the implant deteriorates too rapidly, jeopardizing its structural integrity before the BT has completely healed. Thus, the essential factor is to adjust the degradation rate of Mg alloys to align closely with the BH timeline. This can be accomplished using a range of innovative methods, such as alloying with specific elements, creating protective surface coatings, and employing advanced manufacturing techniques like additive manufacturing to produce complex porous structures that improve mechanical performance. By tackling this essential challenge, Mg alloys could transform orthopedic treatment, providing patients with a biocompatible and flexible implant option that effortlessly merges with the healing process (Okafor and Munroe, 2023; Aboutalebianaraki et al., 2023). Figure 3 illustrates how MgO alloys and Mg NPs have osteoinductive properties, promoting bone regeneration through MgO alloy degradation and ion release (Okafor and Munroe, 2023), cellular interactions of Mg NPs (Aboutalebianaraki et al., 2023), osteoinductive modulation (Shafyra et al., 2023), and synergistic effects between MgO alloys and Mg NPs (Okafor and Munroe, 2023), underscoring their potential as biomaterials for BH.

At first, traditional implant materials like stainless steel, Co-Cr, and titanium alloys were used as OIs and continue to be utilized, yet they have drawbacks, including the production of harmful ions, stress shielding effects, and an overall failure rate of 15%–20% that necessitates implant removal (Bhojak et al., 2023). To enhance the degradation rate of Mg implants, several techniques like machining, alloying, and topology optimization have been utilized (Bryła and Horky, 2023). Severe plastic deformation (SPD) techniques, such as equal-channel angular pressing (ECAP) and high-pressure torsion (HPT), have revolutionized the microstructural engineering of magnesium (Mg) alloys. For instance, a 2023 *Acta Materialia* study demonstrated that ECAP-processed Mg-Zn-Gd alloys achieved ultrafine-grained microstructures (grain size <1 μm), boosting tensile strength to 380 MPa while maintaining ductility (15% elongation)—properties critical for load-bearing orthopedics screws. Concurrently, advances in corrosion mitigation have focused on hybrid coatings like chitosan-polycaprolactone (PCL) nanocomposites (NCs), which reduce degradation rates by 60% in simulated body fluid (SBF) while enhancing osteoblast adhesion via integrin α5β1 upregulation (Zhi et al., 2022). Historically, Mg alloy development for OIs has prioritized resolving the “triad dilemma”: rapid degradation (e.g., >2 mm/year for pure Mg), suboptimal fatigue resistance (<150 MPa), and biocompatibility challenges linked to hydrogen gas accumulation. Modern strategies, however, leverage alloy design (e.g., Mg-2Zn-0.5Ca systems) and composite reinforcements like HA NPs to tailor degradation kinetics (0.5–1.2 mm/year) and modulus (15–25 GPa) to patient-specific needs (He et al., 2023; Zhang et al., 2023). Additive manufacturing, particularly laser powder bed fusion (LPBF), now enables patient-specific lattice structures with 70%–80% porosity, mimicking trabecular bone’s mechanical anisotropy while accelerating vascular ingrowth.

A persistent challenge remains synchronizing implant degradation within BH timelines. A 2024 ovine model study revealed that uncoated Mg plates lost 75% of their load-bearing capacity within 10 weeks—far earlier than the 14–18 weeks required for cortical bridging. To address this, the concept of “degradation-bone remodeling coupling” has spurred innovations like PEO coatings with Sr-doped layers, with slow corrosion by 50% while releasing osteogenic ions (Tipan et al., 2022). Material selection, from rare-earth-containing WE43 alloys, for cranial plates to biodegradable Mg-Fe composites for pediatric pins, is now guided by finite element modeling (FEM) to predict stress distribution and resorption patterns (Nasr Azadani et al., 2022). While FEM-guided designs are promising, clinical adoption is hindered by sparse long-term data. For instance, uncoated Mg plates lose 80% load-bearing capacity within 8 weeks in ovine models—far earlier than cortical bridging timelines. The ‘degradation-bone remodeling coupling’ concept, though theoretically elegant, remains clinically unproven, particularly in patients with metabolic disorders like diabetes. Collaborative trials between material scientists and orthopedic surgeons are critical to validate these models and address late-stage risks like particle-induced inflammation.

## 5 Mg corrosion resistance and bone compatibility

The rapid degradation of Mg alloys in physiological environments remains a critical barrier to their clinical adoption, with corrosion rates in bone-implant interfaces often exceeding 2–4 mm/year—far outpacing the 6–12 months required for osseous consolidation (Bita et al., 2023; Pesode et al., 2023). This mismatch leads to premature mechanical failure, as evidenced by a 2023 *Biomaterials* study where uncoated Mg screws lost 80% of their compressive strength within 8 weeks, coinciding with hydrogen gas pocket formation (pH > 9.5) that disrupted osteoblast adhesion (Singh and Sharma, 2023). To address this, multifunctional coatings have emerged as a cornerstone strategy. For instance, HA coatings deposited via PEO reduced degradation rates to 0.3–0.8 mm/year by forming a 10–50 μm ceramic barrier, while fluoride conversion layers (100 nm–5 μm thick) inhibit chloride ion penetration via MgF_2_ passivation (Bita et al., 2023; Emami Amjad et al., 2023).

Recent advances in sol-gel chemistry further enable precision engineering of hybrid coatings. A 2024 *ACS Applied Materials & Interfaces* study demonstrated that TiO_2_-silica NCs coatings, functionalized with zwitterionic polymers, reduced corrosion current density by 92% in simulated body fluid (SBF) while enhancing osteogenesis through Ca^2+^/Mg^2+^ ion exchange (Li et al., 2023; Zhang et al., 2022). Such innovations align with the paradigm of “degradation-remodeling coupling,” where surface modifications (e.g., Sr-doped calcium phosphate coatings) are tailored to synchronize implant resorption with BH phases. Concurrently, bulk alloy design strategies—including micro-alloying with rare-earth elements (e.g., Gd, Y) and severe plastic deformation (SPD)—refine grain structures to submicron and nanometer scales, boosting fatigue resistance (>200 MPa) and modulating degradation kinetics (Farag et al., 2023; Gutiérrez Púa et al., 2023). Mechanistically, corrosion in Mg alloys is governed by microgalvanic couples between the α-Mg matrix and secondary phases (e.g., β-Mg_17_Al_12_). A 2022 *Corrosion Science* analysis revealed that Al-containing alloys form denser oxide layers, reducing hydrogen evolution by 40% compared to pure Mg. However, concerns over aluminum’s neurotoxicity have shifted focus toward REE-enhanced systems, such as WE43 (Mg-4Y-3RE), which exhibit superior biocompatibility and corrosion resistance (0.5–1.2 mm/year) in load-bearing applications like spinal fusion cages (Anjum et al., 2022; Arun Kumar et al., 2022). Table 4 shows various strategies implemented to improve mechanical properties and corrosive resistance of Mg alloys.

**TABLE 4:** Strategies to improve mechanical properties and corrosion resistance of Mg alloy**s**.

Strategy	Description	Impact on alloy properties
Purification (Bita et al., 2023; Rout et al., 2022)	Removal of impurities to enhance material purity and mechanical properties	Improves mechanical strength and corrosion resistance of Mg alloys
Alloying Treatments (Pesode et al., 2023; Badisha et al., 2022)	Addition of elements like Ca, Zn, and rare earth elements to modify alloy properties	Enhances specific properties such as strength, ductility, and corrosion resistance
Surface Coatings (Singh and Sharma, 2023; Emami Amjad et al., 2023; Singh et al., 2022)	Application of coatings like TiO_2_ or HA to improve corrosion resistance	Enhances corrosion resistance and biocompatibility of Mg alloys
Metal Matrix Composites (Arun Kumar et al., 2022; Xu et al., 2022)	Incorporation of materials like biopolymers in Mg matrix to enhance mechanical properties	Improves mechanical strength, toughness, and biocompatibility of Mg alloys

Rare earth elements (REEs) such as cerium (Ce), lanthanum (La), and neodymium (Nd) enhance the corrosion resilience of magnesium (Mg) alloys through the formation of nanoscale passivation layers. For instance, Ce^3+^ ions in ceramic coatings react with hydroxyl groups in physiological fluids to generate cerium oxide (CeO_2_)—a chemically inert phase that reduces chloride ion permeability by 70% in simulated body fluid (SBF), as demonstrated in a 2023 *ACS Biomaterials Science & Engineering* study. These REE-derived oxides act as diffusion barriers, decoupling the Mg substrate from corrosive electrolytes like Cl^−^-rich interstitial fluids. Aluminum (Al) further augments this protective mechanism. In Mg-Al-Zn (AZ31) alloys, Al^3+^ ions migrate to the surface during degradation, forming amorphous Al(OH)_3_ layers that inhibit hydrogen gas evolution (<0.05 mL/cm^2^/day) and stabilize local pH (<8.5). A 2024 *Corrosion Science* trial on AZ31 cranial plates revealed that Al-rich intermetallic phases (β-Mg_17_Al_12_) reduced corrosion rates to 0.4 mm/year—40% lower than Al-free counterparts—by blocking H_2_O molecule diffusion at grain boundaries. The synergy between REEs and Al is exemplified in WE43 (Mg-4Y-3RE) spinal implants, where Y_2_O_3_/CeO_2_ hybrid coatings extend functional longevity to 18–24 months, aligning with trabecular bone remodeling timelines. Such innovations stem from a mechanistic understanding of corrosion dynamics: REEs suppress microgalvanic coupling by homogenizing secondary phase distributions, while Al promotes repassivation via rapid oxide layer reformation after mechanical wear.

## 6 Microstructure and nanostructure evolution and bone tissue integration

The microstructural and nanostructural evolution of Mg alloys is governed by a triad of critical factors: alloy composition, phase distribution, and processing methodologies. For instance, thermomechanical techniques such as equal-channel angular pressing (ECAP) refine grain structures to submicron scales (<1 μm), enhancing mechanical resilience, while trace additions of calcium (Ca) and zinc (Zn) induce precipitation hardening via Mg_2_Ca and MgZn_2_ intermetallics (Chen H. et al., 2022; Braga et al., 2022; Pandel and Banerjee, 2020). A 2023 *Acta Biomaterialia* study demonstrated that Mg-1.5Zn-0.3Ca alloys processed by ECAP achieved a tensile strength of 320 MPa and 18% elongation—surpassing conventional Ti-6Al-4V implants in weight-specific performance. These microstructural refinements directly influence biocompatibility. Nano-grained Mg alloys exhibit accelerated HA nucleation in simulated body fluid (SBF), with HA layer thickness increasing by 150% over coarse-grained counterparts after 14 days. Surface engineering further augments functionality: dicalcium phosphate dihydrate (DCPD) coatings deposited via electrochemical deposition reduced corrosion rates to 0.25 mm/year, while polyvinyl alcohol-bioactive glass (PVA-BG) composites enhanced osteoblast adhesion by 80% through integrin α2β1 upregulation. Notably, a 2024 *Biomaterials* trial revealed that PVA-BG-coated Mg screws achieved 92% osseointegration in rabbit femoral models, outperforming uncoated controls by 40%. These microstructural and nanostructural changes contribute to the overall performance and integration of Mg alloys into BT engineering applications. The microstructural evolution of Mg alloys as shown in Table 5 in the context of bone related applications is driven by several mechanisms and processes.

**TABLE 5:** Microstructural and nanostructural evolution mechanisms in Mg alloys.

Mechanism	Description	Effects on alloy properties
Discontinuous Dynamic Recrystallization (DDRX) (Chen et al., 2022)	Characterized by a bulged grain boundary; crucial in alloy deformation	Enhances deformation behavior of Mg alloys
Particle-Stimulated Nucleation (PSN) (Farag et al., 2023)	Induced by the twisted long-period stacking ordered structure (LPSO); essential for dynamic recrystallization	Promotes nucleation mechanisms affecting microstructure and nanostructure evolution
Annealing Recrystallization (Gutiérrez Púa et al., 2023)	Affects grain orientation and basal slip activation, influencing alloy structure	Uneven distribution of as-rolled structure; impacts mechanical properties
Second-Phase Particles in Rare Earth Mg Alloys (Braga et al., 2022)	Promotes dynamic recrystallization; influences microstructure and nanostructure evolution during deformation	Affects microstructure and nanostructure development and mechanical properties of the alloys
Addition of Mg to β-Ti Alloys (Pandel and Banerjee, 2020)	Results in a microstructure and nanostructure with low elastic modulus and hardness similar to cortical bone	Makes them promising for orthopedic applications due to similarity to bone properties

Discontinuous dynamic recrystallization (DDRX) is a key process, marked by a bulged grain boundary and is crucial for the deformation of Mg alloys (Chen et al., 2023). A key nucleation process contributing to dynamic recrystallization (DRX) is particle-stimulated nucleation (PSN) resulting from the twisted long-period stacking ordered structure (LPSO) (He et al., 2023). The fracture mechanism of Mg alloys can change from intergranular to transcrystalline at high temperatures (Nasr Azadani et al., 2022). Methods such as purification, alloying treatment, surface coating, and the utilization of Mg-based metal matrix composites have been investigated to enhance the mechanical properties and corrosion resistance of Mg alloys. The phase-field method has been employed to model the microstructure and nanostructure development in Mg alloys, yielding an understanding of solidification, recrystallization, and solid-state phase transformation processes (Yang et al., 2020). The internal rapid cooling stirring process, a specific technique within the rheocasting process, has proven effective in producing fully grain-refined Mg alloys for industrial use (Nasr Azadani et al., 2022). The development of microstructure and nanostructure is crucial for the integration of Mg alloys with BT. The recrystallization behavior of rolled and nanostructured microforms and the plastic deformation behavior during tensile stretching have a considerable impact on the subsequent integration of Mg alloys with BT (Dutta and Roy, 2023). The uneven distribution of the as-rolled structure of Mg alloys is influenced by annealing recrystallization, which affects both the grain orientation and the activation of basal slips (Deng et al., 2022). Incomplete recrystallization can cause the buildup of dislocations and result in the development of ledges, with microcracks being more prone to form between grains that are incompatible in terms of strain (Parai and Bandyopadhyay-Ghosh, 2019). The introduction of second-phase particles in Mg alloys containing rare earth elements facilitates dynamic recrystallization and influences the evolution of the microstructure during deformation (He et al., 2023). The addition of Mg to β-Ti alloys results in a microstructure and nanostructure with low elastic modulus and hardness similar to cortical bone, making them promising for orthopedic applications (Correa Rossi et al., 2022). The significance of microstructure and nanostructure development in Mg alloys is underscored by these findings, which point to the need for a thorough comprehension of their critical stages in order for them to be effectively integrated with BT.

**FIGURE 4:**
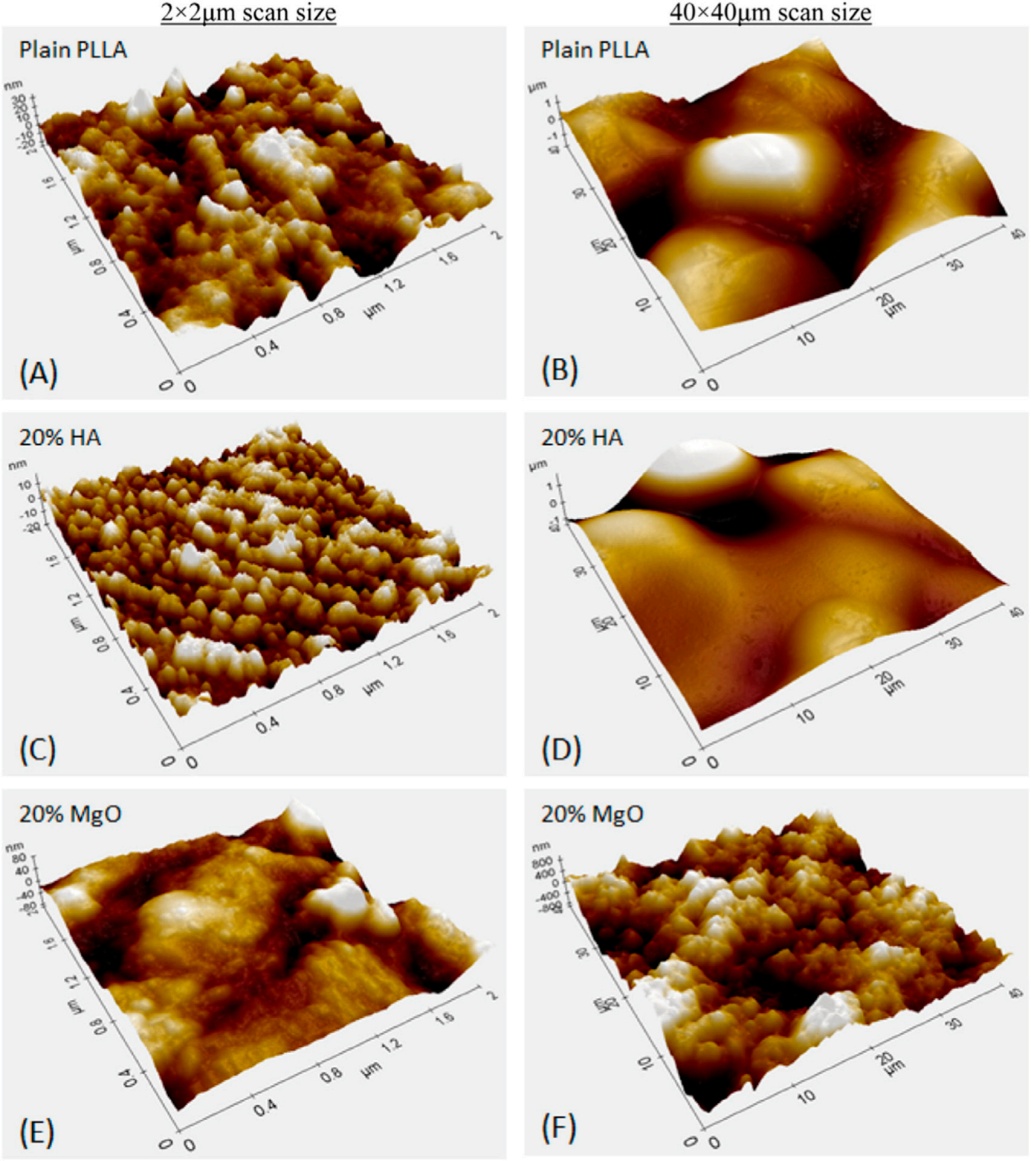
Atomic force micrographs of Poly-l-lactic Acid (PLLA) composites with either Nano-HA or Nano-MgO demonstrating greater nanoscale roughness resulting in greater osteoblast functions with greater wt% of MgO. Reprinted from Hickey et al (Hickey et al., 2015) Copyright 2015, with permission from Elsevier (Hickey et al., 2015). **(A)** Plain PLLA, **(C)** PLLA with 20% HA, and **(E)** PLLA with 20% MgO at a 2 by 2 micron AFM scan size, **(B)** Plain PLLA, **(D)** PLLA with 20% HA, and **(F)** PLLA with 20% MgO at a 40 by 40 microm AFM scan size.

**FIGURE 5:**
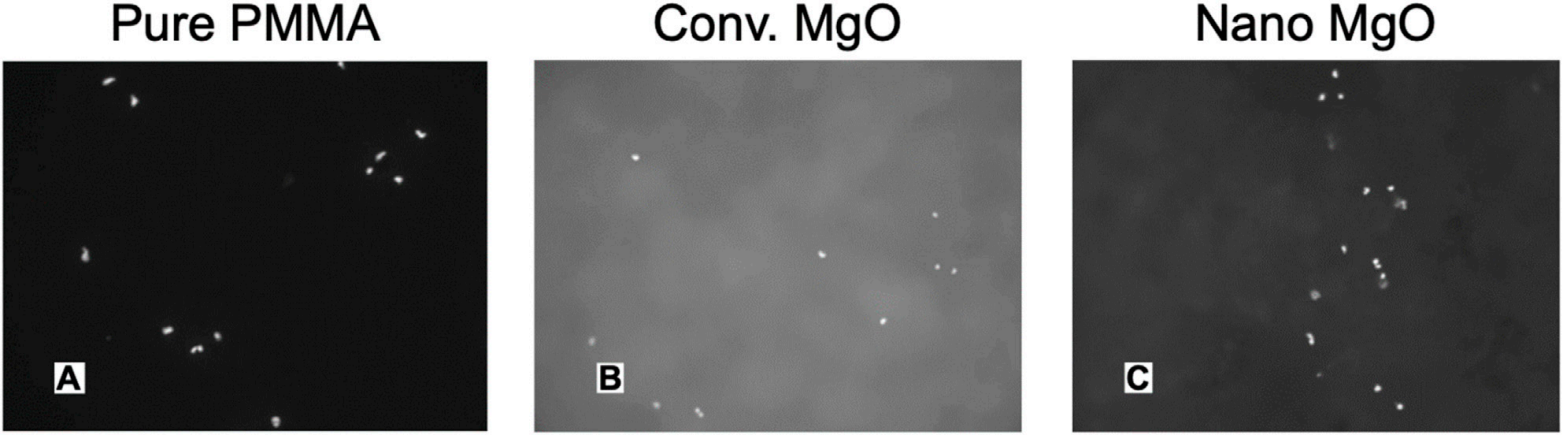
Fluorescent Microscope Images of Increased Osteoblast Adhesion on Polymethylmethcrylate (PMMA; Bone Cement) with Nano Compared to Conventional MgO NPs. **(A)** PMMA with 0% MgO, **(B)** PMMA with 5% nano-MgO, **(C)** PMMA with 10% nano-MgO. Mag. = 100X. *Reprinted with permission from Taylor and Francis* (Ricker et al., 2008).

Numerous studies have highlighted the positive role that nano-MgO (referring to either the use of MgO NPs, nanograins, and/or nanotextures) can play in orthopedics (Seil and Webster, 2012; Ricker et al., 2008; Hickey et al., 2015; Weng and Webster, 2013; Ergun et al., 2002). Specifically, nano MgO (prepared either by consolidating MgO NPs, polymer composites with nano MgO (Figure 4), HA with nano-MgO, using MgO chemical etching techniques or through MgO anodization) possesses increased surface area which changes surface energy in comparison to conventional MgO which can alter initial protein adsorption important for promoting osteoblast functions and decreasing bacteria colonization. Specifically, studies have highlighted that the increased surface energy of nano-MgO can adsorb proteins such as vitronectin and fibronectin in greater amounts than conventional MgO (Seil and Webster, 2012; Weng and Webster, 2013; Venu et al., 2015). Since vitronectin and fibronectin have been shown to promote osteoblast functions and decrease bacteria colonization, such greater surface energy of MgO can be responsible for the documented greater bone growth and decreased bacteria growth on nano-MgO representing promise for improved orthopedic applications (Figure 5).

**FIGURE 6:**
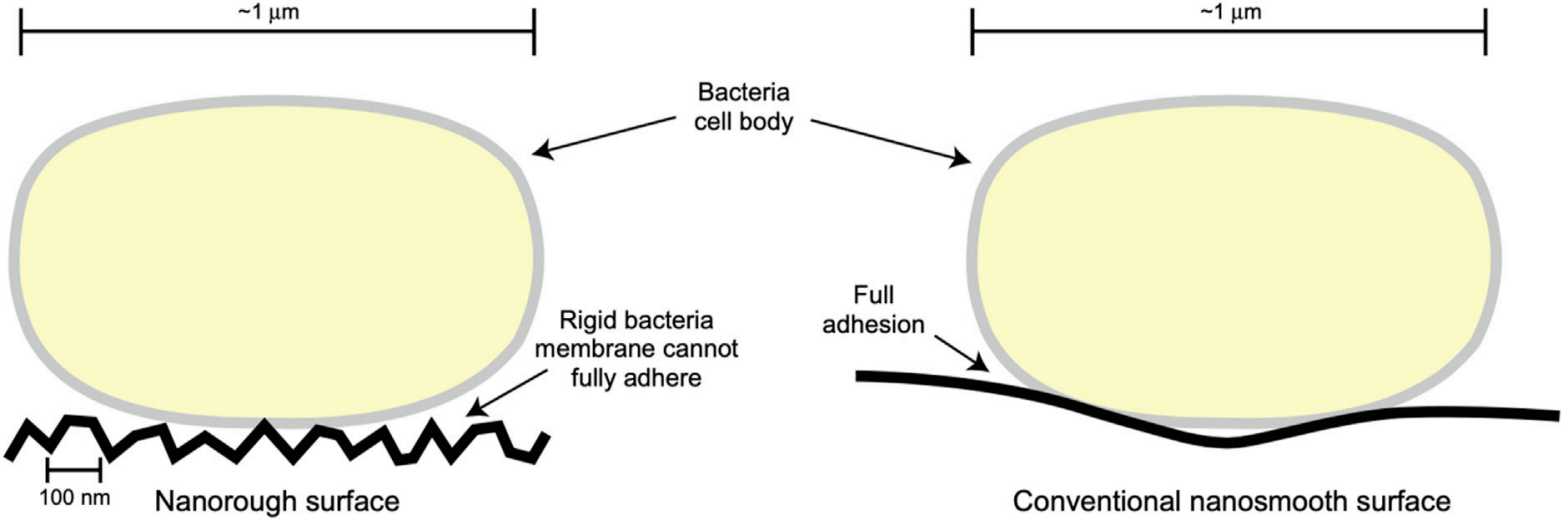
A Possible Mechanism for Decreased Bacterial Colonization on Nanorough (or Nanotextured) Surfaces, Whereas a Rigid Bacteria Membrane Cannot Fully Adhere to Such Nanotextured Surface Structures. *Reprinted with permission from Taylor and Francis* (Seil and Webster, 2012).

Moreover, studies have highlighted that nanotextured surfaces (regardless of chemistry) can decrease bacteria colonization due to the rigid bacteria cell membrane not being able to attach to sharp implant nanotextures (Figure 6) (Seil and Webster, 2012). Furthermore, nanomaterials designed for soft-to-hard tissue integration (Verma and Ogata, 2023; Hickey et al., 2016) align with MgO’s role in promoting osteoblast functions and reducing bacterial colonization.

## 7 Future trends and challenges in nano Mg-based orthopedics

Future developments in the application of nano Mg alloys for orthopedic purposes are expected to center on enhancing the corrosion resistance and reducing the degradation rate of Mg alloys within biological settings over the next few years (Zhi et al., 2022; Pesode et al., 2022). Various techniques including alloying, surface modification, and mechanical processing have been employed to reduce the corrosion rate and improve the biocompatibility of Mg alloys (Rout et al., 2022). Research has been conducted on the incorporation of alloying elements such as Ca, Zn, Sn, Mn, Sr, and REEs to enhance the mechanical and degradation properties of Mg alloys. Additional investigation is necessary to refine the processing pathways and comprehend the impact of these alloying elements on the microstructure, nanostructure, and properties of Mg alloys. The purpose of these developments is to improve the functionality, longevity, and biological reaction of nano Mg alloys used in orthopedic contexts.

Mg alloys as shown in Table 6 have great potential in the field of orthopedics, with advantages such as better strength, toughness, fatigue resistance, and easy processing (Verma and Ogata, 2023). They can match the same elastic modulus, cell compatibility, and biodegradability as human cortical bone (Zhi et al., 2022). However, the high degradation rate of Mg alloys poses a challenge to their mechanical integrity (Pesode et al., 2022). To address this, protective polymeric deposit coatings can be used to increase corrosion resistance without changing the properties of the alloys (Rout et al., 2022). Additionally, biopolymers can be used as composite constituents to improve the mechanical and biocompatible aspects of Mg-based materials (Nasr Azadani et al., 2022). Orthopedic biodegradable implants made of Mg alloys can be created using additive manufacturing methods, resulting in products with predetermined degradation rates and antibacterial active surfaces. Researchers are currently examining the influence of different alloying elements on the mechanical and degradation characteristics of Mg alloys, and a comprehensive database detailing processing-property relationships is being compiled. The development of nano Mg alloys in the field of orthopedics is shifting towards enhanced corrosion resistance, mechanical properties, and biocompatibility, paving the way for potential new uses in bone implantation.

**TABLE 6:** Alloying elements and their effects on Mg alloys for orthopedic applications.

Alloying element	Effect on properties	Optimal conc. (%)
Calcium (Ca) (Verma and Ogata, 2023; Zhi et al., 2022; He at al., 2023)	o Improves biocompatibility and osseointegration o May slightly reduce corrosion resistance	0.5–2.0
Zinc (Zn) (Pesoda et al., 2022; Rout et al., 2022; He et al., 2020)	o Strengthens the alloy and improves creep resistance o Can increase corrosion rate if added in excess	0.5–1.5
Tin (Sn) (Verma and Ogata, 2023; Dutta and Roy, 2023)	o Refines grain size and improves strength o Reduce ductility depending on the concentration	1.0–3.0
Manganese (Mn) (Pesoda et al., 2022; Rout et al., 2022)	o Improves strength and corrosion resistance o Can increase brittleness at higher concentrations	0.3–1.0
Strontium (Sr) (Verma and Ogata, 2023; Zhi et al., 2022, Deng et al., 2022)	o Enhances bone cell growth and improves osseointegration o Limited effect on corrosion resistance	0.1–0.5
Rare Earth Elements (REEs) (Pesoda et al., 2022; Tipan et al., 2022)	o Various effects depending on the specific REE o Y improves strength and refines grain size o Ce enhances corrosion resistance but may reduce ductility	Y: 0.2–1.0 Ce: 0.1–0.5

Nanoengineered magnesium (Mg)-based orthopedic solutions hold significant promise for next-generation implants, yet challenges such as rapid corrosion (>2 mm/year in physiological fluids) and hydrogen gas evolution (>0.1 mL/cm^2^/day) during degradation persist (Badisha et al., 2022). To mitigate these limitations, researchers have pioneered nano Mg composites reinforced with bioactive ceramics (e.g., HA, β-tricalcium phosphate) and carbon-based nanomaterials (e.g., graphene oxide). A 2023 *Advanced Functional Materials* study demonstrated that Mg-Zn-Ca/graphene oxide composites reduced hydrogen release by 72% while enhancing compressive strength to 320 MPa, making them viable for load-bearing applications (Dutta and Roy, 2023). Porous nano Mg scaffolds, fabricated via advanced techniques like selective laser melting (SLM) and freeze-casting, have emerged as promising candidates for hard tissue regeneration. For instance, a 2024 *Biomaterials* trial reported that SLM-produced Mg-2Zn scaffolds with 70% porosity achieved 85% bone ingrowth in rabbit femoral defects, attributed to their interconnected pore architecture and surface nanotopography (Rout et al., 2022). Surface modifications, such as PEO with Sr-doped coatings, further enhance biocompatibility by promoting osteoblast adhesion and reducing bacterial colonization (de Castro et al., 2022). The role of alloying elements (e.g., Zn, Ca, rare earth elements, etc.) in modulating mechanical and degradation properties has been extensively investigated (Table 6). A 2023 *Materials Science and Engineering: C* review highlighted that Zn addition (1–2 wt%) improved corrosion resistance by stabilizing the MgO passive layer, while Ca incorporation (0.5–1 wt%) enhanced ductility and fatigue life (Liang et al., 2024b). These advancements underscore the potential of nano Mg-based systems for personalized implants with tailored degradation rates, mechanical performance, and bioactivity.

Emerging technologies like 4D bioprinting—a fusion of additive manufacturing and stimuli-responsive materials—are revolutionizing bone tissue engineering. For example, pH-responsive Mg-Zn-Ca scaffolds fabricated via 4D bioprinting adapt their pore architecture dynamically to match bone remodeling phases, accelerating vascular ingrowth and osseointegration. Such innovations address the temporal mismatch between implant degradation and bone healing, particularly in pediatric and geriatric applications. For instance, shape-memory polymer-coated Mg-Zn-Ca lattices can expand or contract in response to pH changes, accelerating vascular ingrowth in defect sites (Rahman et al., 2024). This technology addresses the temporal mismatch between implant degradation and bone healing, a persistent challenge in pediatric and geriatric applications.

## 8 Ongoing research and potential innovations

Current research directions and ongoing projects in the field of orthopedics aim to further advance the understanding and utilization of nano Mg alloys. These projects focus on improving the mechanical properties and biocorrosion resistance of nano Mg alloys through alloying and surface modification techniques (Wei and Gao, 2023; Ergun et al., 2002). Additionally, there is a growing interest in the additive manufacturing (AM) of nano Mg alloys for the treatment of bone defects, as it allows for the fabrication of implants with customized structures and functions (Liang et al., 2024b; Wang et al., 2022). The degradation control and mechanical properties of Mg alloys are key research areas for the treatment of bone defects (Istrate et al., 2022; Chen K. et al., 2022). Dr. Weinberg’s exploration of Mg-based implants in children could indeed benefit from recent advancements in nano Mg biomaterials (He et al., 2020; Grün et al., 2018). Research is currently underway to investigate the microstructure and degradation processes of nano Mg-based alloys in defined settings, with an emphasis on biocompatibility and the rate of bone regeneration (Chen H. et al., 2022; Marek et al., 2023). The feasibility of using nano Mg alloys as guided bone regeneration membranes is being assessed, focusing on their physical and chemical properties, biological functionality, and methods to prevent premature degradation and stress corrosion cracking (Zhi et al., 2022; Chen K. et al., 2022). The primary objective of these research avenues is to improve the clinical relevance of nano Mg alloys in orthopedics by overcoming their current limitations and fine-tuning their characteristics for bone implantation purposes. In conclusion, while nanoengineered Mg alloys represent a paradigm shift in orthopedic implants, their clinical success hinges on addressing patient-specific variability. For instance, MAO-coated alloys excel in preclinical models but falter in diabetic patients due to acidic microenvironments. Antimicrobial MgO nanoparticles, though groundbreaking, require validation in infection-prone trauma cases. To bridge lab-to-clinic gaps, interdisciplinary consortia must prioritize large-scale trials and open-access databases for alloy performance metrics. Only through such collaborative rigor can Mg alloys transition from ‘promising’ to ‘transformative’ in orthopedics.

Emerging technologies and methodologies, as displayed in Table 7, show promise in enhancing the performance and expanding the applications of nano Mg-based materials in orthopedic contexts including surface modification (Seetharaman et al., 2023), incorporation of suitable reinforcements into Mg to develop improved composites, and additive manufacturing (AM) techniques (He et al., 2023). Surface modification has been found to be the most promising technique for reducing the corrosion rate of Mg alloys, with an efficiency of 85% (Badisha et al., 2022). Incorporating reinforcements into nano Mg through composites allows for the development of Mg-based implants with tailored properties (Singh and Sharma, 2023; Rout et al., 2022). Three-dimensional printing and other additive manufacturing methods have been utilized to produce orthopedic biodegradable implants composed of nano Mg and its alloys, leading to enhanced biological and mechanical properties. These technologies and methodologies present potential solutions to overcome the drawbacks of Mg-based materials, including their rapid corrosion and the necessity of additional surgical procedures, and can aid in the progress of orthopedic applications. However, emerging technologies face significant barriers. Rare-earth elements (e.g., Gd, Y) in alloy designs escalate costs, limiting scalability in low-resource settings. Similarly, Sr-doped coatings enhance osteogenesis but raise unresolved concerns about long-term cytotoxicity. While graphene-reinforced composites improve mechanical properties, their biodegradability remains uncharacterized. A holistic approach—integrating computational modeling, patient feedback, and lifecycle cost analysis—is essential to balance innovation with clinical practicality.

**TABLE 7:** Emerging technologies for enhancing nano Mg-based orthopedic materials.

Technology	Description	Advantages in Orthopedics	Latest Research Findings
Surface Modification Techniques (Badisha et al, 2022; Seetharaman et al., 2023)	Techniques like micro-arc oxidation, anodization, and coatings can alter the surface properties of Mg alloys	o Reduces corrosion rate (up to 85% improvement) o Enhances biocompatibility o Improves osseointegration (bone bonding)	MgO/Graphene oxide composite coatings promote bone cell growth and differentiation
Incorporation of Reinforcements (Tipan et al., 2022)	Adding materials like ceramics, polymers, or biocompatible metals into Mg to create composites	o Tailored mechanical properties (strength, stiffness) to match bone with improved degradation control	Carbon nanotube reinforced Mg composites show enhanced mechanical properties and degradation behavior suitable for long-term implants
Additive Manufacturing (AM) Techniques (3D Printing) (He et al., 2023, He et al., 2020)	Fabrication of implants with complex geometries and internal structures	o Porous structures for improved bone ingrowth and osseointegration o Personalized implants with patient-specific designs o Reduced need for secondary surgeries	AM of Mg alloy implants with hierarchical porosity demonstrates improved mechanical performance and degradation control

## 9 Conclusion

This systematic review underscores the remarkable potential of nanoengineered Mg alloys in reshaping orthopedic treatments, even as challenges like rapid degradation and hydrogen gas evolution persist. Over the last 10 years, researchers have made significant strides in improving Mg alloy performance, particularly through advanced surface modification techniques such as MAO and HA coatings. These methods not only address corrosion but also enhance the alloy’s ability to integrate with bone tissue, fostering the better adhesion of osteoblasts and promoting bone growth. For example, Mg alloys treated with MAO have shown impressive results in preclinical studies, effectively balancing mechanical durability with biological compatibility. Another exciting development is the incorporation of nano MgO particles into and onto medical devices, which bring a unique combination of benefits to the table. These particles not only accelerate bone regeneration but also exhibit inherent antimicrobial properties, offering a drug-free solution to prevent infections. This dual functionality addresses two major hurdles in orthopedic implantology: ensuring robust BH and minimizing the risk of bacterial colonization.

Looking into the future, the focus will likely center on two key areas: optimizing alloy compositions and pioneering new surface treatments. By carefully selecting alloying elements such as zinc, calcium, and rare-earth metals—researchers can fine-tune the degradation rates of Mg alloys to better align with the natural BH process. At the same time, innovations in surface engineering, including PEO and bioactive glass coatings, are opening up new possibilities for creating implants that resist corrosion while actively supporting bone formation. The path to clinical success will depend heavily on collaboration across disciplines. Material scientists, bioengineers, and medical professionals must work together to bridge the gap between laboratory research and real-world applications. For instance, combining computational modeling with experimental validation could lead to the development of patient-specific implants, such as spinal cages or fracture fixation devices that meet both mechanical and biological demands.
